# The genome sequence of a tephritid fruit fly, *Merzomyia westermanni *Meigen 1826

**DOI:** 10.12688/wellcomeopenres.22873.1

**Published:** 2024-08-14

**Authors:** Steven Falk, Liam M. Crowley, Nathan C. Medd, University of Oxford and Wytham Woods Genome Acquisition Lab, Darwin Tree of Life Barcoding collective, Wellcome Sanger Institute Tree of Life Management, Samples and Laboratory team, Wellcome Sanger Institute Scientific Operations: Sequencing Operations, Wellcome Sanger Institute Tree of Life Core Informatics team, Tree of Life Core Informatics collective, Darwin Tree of Life Consortium

**Affiliations:** 1 Independent researcher, Kenilworth, England, UK; 2 University of Oxford, Oxford, England, UK; 3 The University of Edinburgh, Edinburgh, Scotland, Canada

**Keywords:** Merzomyia westermanni, tephritid fruit fly, genome sequence, chromosomal, Diptera

## Abstract

We present a genome assembly from an individual *Merzomyia westermanni* (a tephritid fruit fly; Arthropoda; Insecta; Diptera; Tephritidae). The genome sequence spans 986.20 megabases. Most of the assembly is scaffolded into 6 chromosomal pseudomolecules. The mitochondrial genome has also been assembled and is 19.45 kilobases in length. Gene annotation of this assembly on Ensembl identified 25,765 protein-coding genes.

## Species taxonomy

Eukaryota; Opisthokonta; Metazoa; Eumetazoa; Bilateria; Protostomia; Ecdysozoa; Panarthropoda; Arthropoda; Mandibulata; Pancrustacea; Hexapoda; Insecta; Dicondylia; Pterygota; Neoptera; Endopterygota; Diptera; Brachycera; Muscomorpha; Eremoneura; Cyclorrhapha; Schizophora; Acalyptratae; Tephritoidea; Tephritidae; Tephritinae; Eutretini; *Merzomyia*; *Merzomyia westermanni* Meigen 1826 (NCBI:txid2795681).

## Background

**Figure 1.**
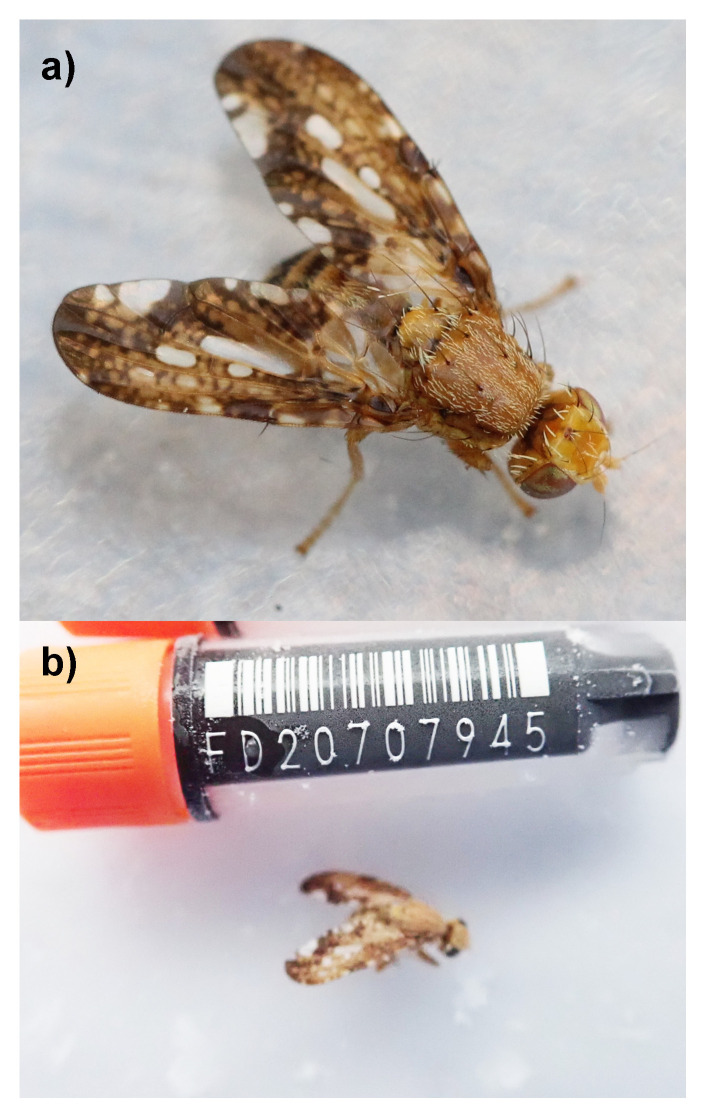
Photographs of *Merzomyia westermanni*: **a**) *M. westermanni* adult (© Marie Lou Legrand, 2015) https://www.inaturalist.org/photos/92418977 **b**) The specimen used for genome sequencing (idMerWest1).

*Merzomyia westermanni* Meigen 1826, is a medium-sized picture-winged fruit fly belonging to the family Tephritidae, also commonly known as the ‘Swiss Cheese Tephritid’. Its wings, which span up to 7.1 mm ( White, 1988), are intricately patterned with reticulated golden-brown markings ( Figure 1a). A distinct clear patch of irregular size and shape is displayed in the centre of each wing. It possesses two pairs of strong dorsal setae on the thorax, dark orange legs, and almost-black oviscape and lower abdomen.

Records of *M. westermanni* span the temperate areas of the western and central Palaearctic, from Yorkshire, UK in the north-west ( Coldwell, 2000) to the Caucasus in the South, with the most easterly records coming from Ukraine ( GBIF.org, 2024). Records in the UK are scattered and restricted largely to the south, but no scarcity status was assigned by Falk *et al*. (2016).

This species is known to be closely associated with *Sencio* spp *.* (Ragwort) especially *S. erucifolius* (Hoary Ragwort), although *S. jacobaea* (Common Ragwort) acts as an additional suitable host ( Seguy, 1934). Larvae develop and pupate in the capitulum of hosts, forming a gall with protruding groups of pappus hairs. Although assumed to be univoltine, the overwintering stage of *M. westermanni* is yet to be confirmed, and most adult records fall between June and September ( GBIF.org, 2024). One record of adult flies performing characteristic wing-waving displays suggested a potential association with, or at least adult preference for, Common Fleabane ( *Pulicaria dysenterica*) ( Falk, 1991).

This species, being a natural parasite of *Sencio spp.*, may be under pressure in areas of intense grazing livestock management. Ragworts, listed as ‘injurious weeds’ in the UK by the Weeds Act, 1959, are toxic if ingested by mammals ( Miranda *et al.*, 1980; Watt, 1987) and are often removed from pastureland. There is ongoing debate about the proper management of Ragwort in pasture, its potential conservation value and control. However, little work has been dedicated to the potential control value of one of Ragwort’s most charismatic inhabitants, the Swiss Cheese Tephritid; *Merzomyia westermanni*.

## Genome sequence report

The genome of an adult *Merzomyia westermanni* ( Figure 1b) was sequenced using Pacific Biosciences single-molecule HiFi long reads, generating a total of 17.85 Gb (gigabases) from 1.55 million reads, providing approximately 32-fold coverage. Primary assembly contigs were scaffolded with chromosome conformation Hi-C data, which produced 138.74 Gbp from 918.84 million reads, yielding an approximate coverage of 141-fold. Specimen and sequencing information is summarised in Table 1.

**Table 1.** Specimen and sequencing data for *Merzomyia westermanni*.

Project information			
**Study title**	Merzomyia westermanni		
**Umbrella BioProject**	PRJEB48049		
**Species**	*Merzomyia westermanni*		
**BioSample**	SAMEA7746463		
**NCBI taxonomy ID**	2795681		
Specimen information			
**Technology**	**ToLID**	**BioSample ** **accession**	**Organism part**
**PacBio long read sequencing**	idMerWest1	SAMEA7746538	Thorax and abdomen
**Hi-C sequencing**	idMerWest1	SAMEA7746537	Head
**RNA sequencing**	idMerWest2	SAMEA113425542	Whole organism
Sequencing information			
**Platform**	**Run accession**	**Read count**	**Base count (Gb)**
**Hi-C Illumina NovaSeq 6000**	ERR7113555	9.19e+08	138.74
**PacBio Sequel IIe**	ERR7123971	1.30e+06	15.98
**PacBio Sequel IIe**	ERR7123972	1.55e+06	17.85
**Chromium Illumina NovaSeq 6000**	ERR7113551	1.37e+08	20.69
**Chromium Illumina NovaSeq 6000**	ERR7113552	1.27e+08	19.13
**Chromium Illumina NovaSeq 6000**	ERR7113554	1.21e+08	18.31
**Chromium Illumina NovaSeq 6000**	ERR7113553	1.67e+08	25.26
**RNA Illumina NovaSeq 6000**	ERR12321224	5.70e+07	8.61

**Figure 2.**
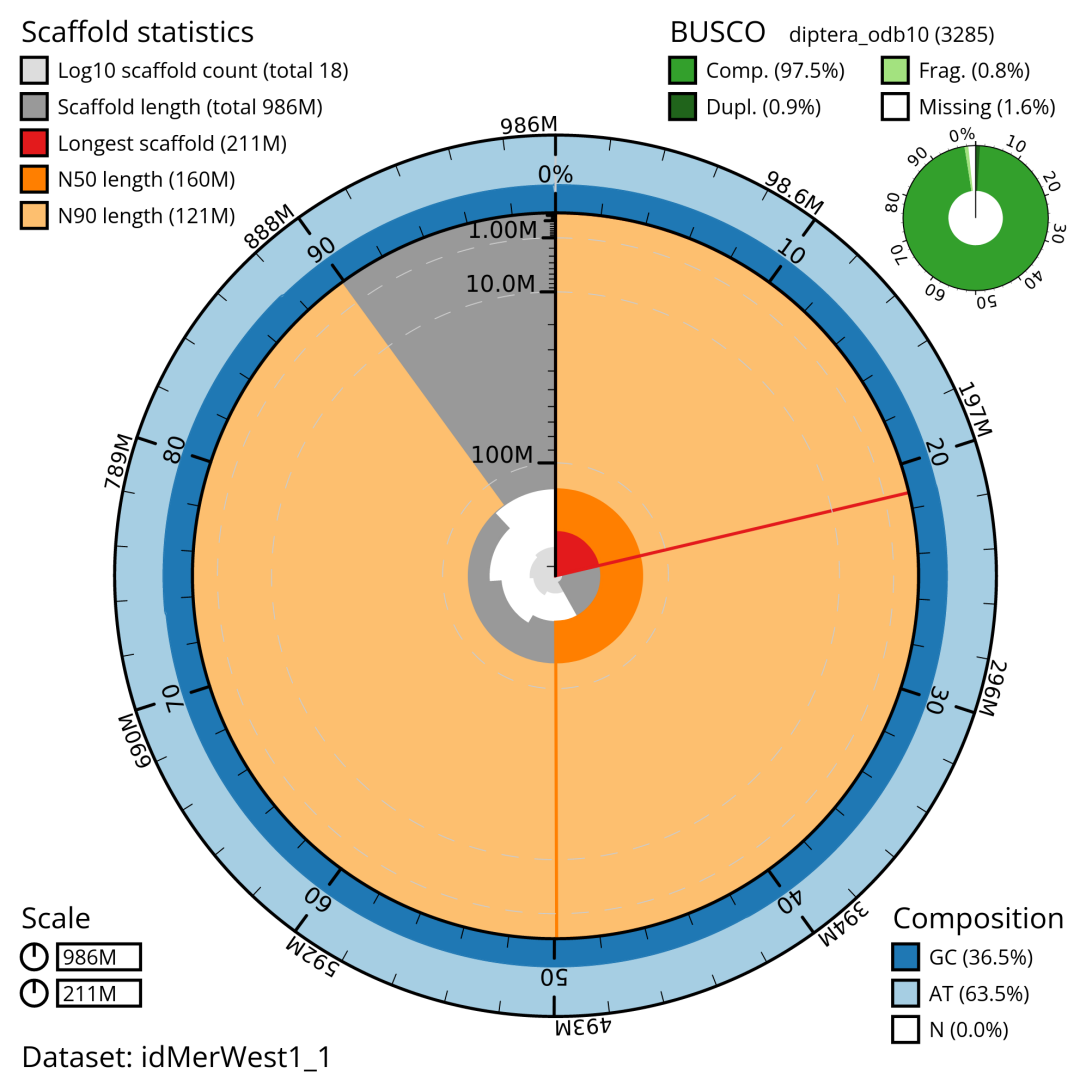
Genome assembly of *Merzomyia westermanni*, idMerWest1.1: metrics. The BlobToolKit snail plot shows N50 metrics and BUSCO gene completeness. The main plot is divided into 1,000 size-ordered bins around the circumference with each bin representing 0.1% of the 986,173,515 bp assembly. The distribution of scaffold lengths is shown in dark grey with the plot radius scaled to the longest scaffold present in the assembly (210,654,766 bp, shown in red). Orange and pale-orange arcs show the N50 and N90 scaffold lengths (160,295,369 and 121,242,192 bp), respectively. The pale grey spiral shows the cumulative scaffold count on a log scale with white scale lines showing successive orders of magnitude. The blue and pale-blue area around the outside of the plot shows the distribution of GC, AT and N percentages in the same bins as the inner plot. A summary of complete, fragmented, duplicated and missing BUSCO genes in the diptera_odb10 set is shown in the top right. An interactive version of this figure is available at https://blobtoolkit.genomehubs.org/view/idMerWest1_1/dataset/idMerWest1_1/snail.

**Figure 3.**
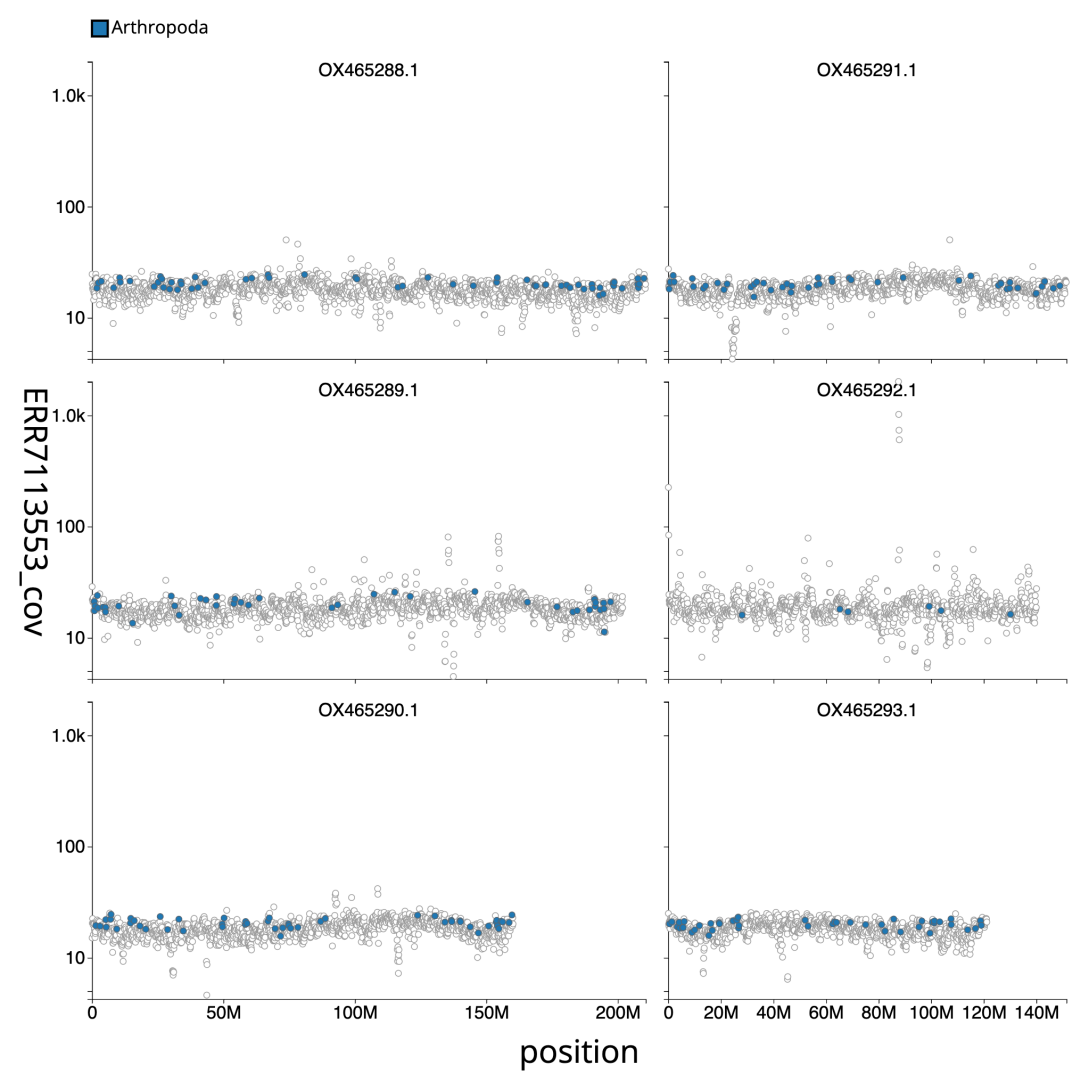
Genome assembly of *Merzomyia westermanni*, idMerWest1.1. Distribution plot of base coverage in ERR7113553 against position for sequences in assembly idMerWest1_1. Windows of 100kb are coloured by phylum. The assembly has been filtered to exclude sequences with length < 2,550,000. An interactive version of this figure is available here.

**Figure 4.**
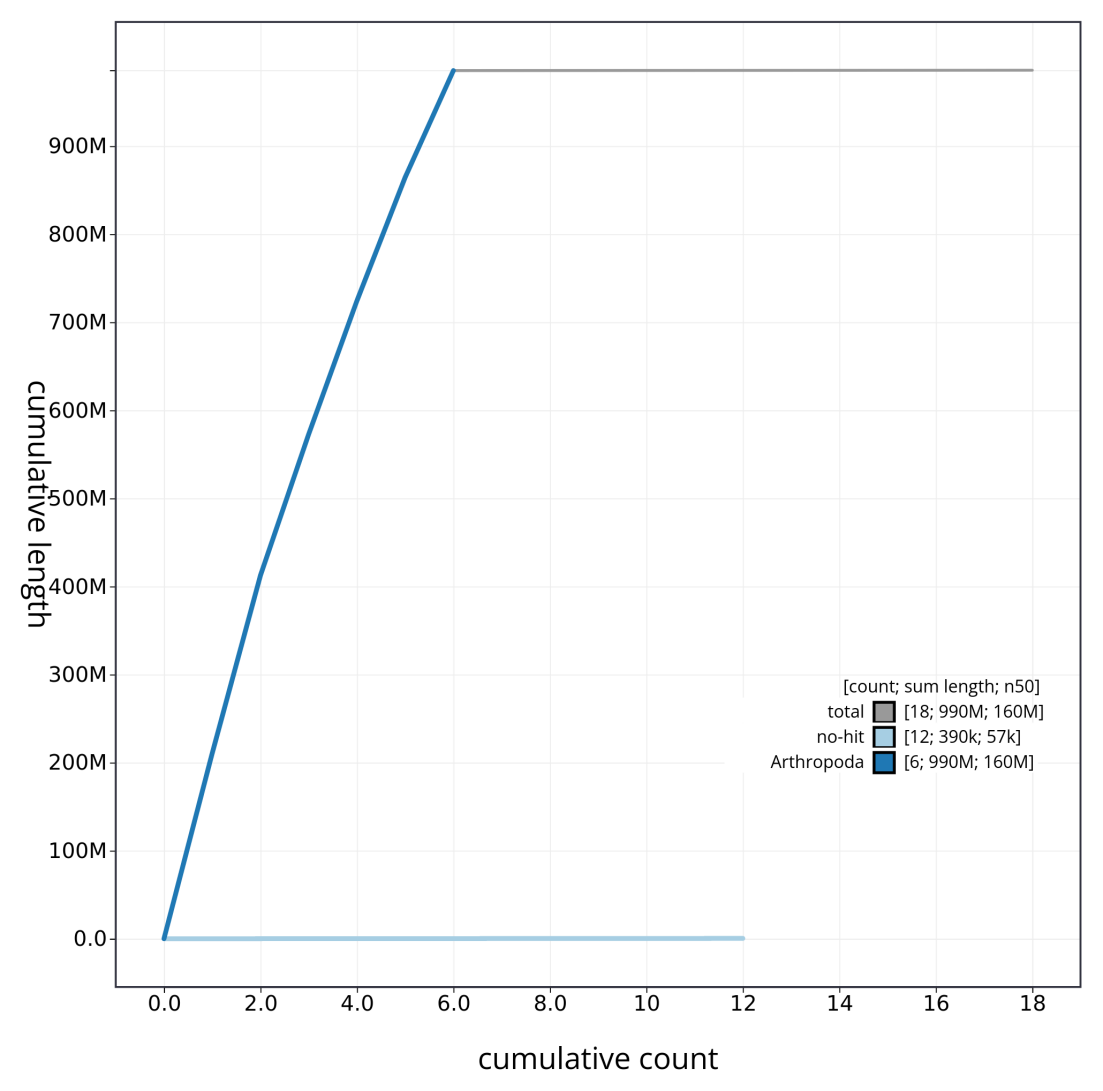
Genome assembly of *Merzomyia westermanni* idMerWest1.1: BlobToolKit cumulative sequence plot. The grey line shows cumulative length for all sequences. Coloured lines show cumulative lengths of sequences assigned to each phylum using the buscogenes taxrule. An interactive version of this figure is available at https://blobtoolkit.genomehubs.org/view/idMerWest1_1/dataset/idMerWest1_1/cumulative.

**Figure 5.**
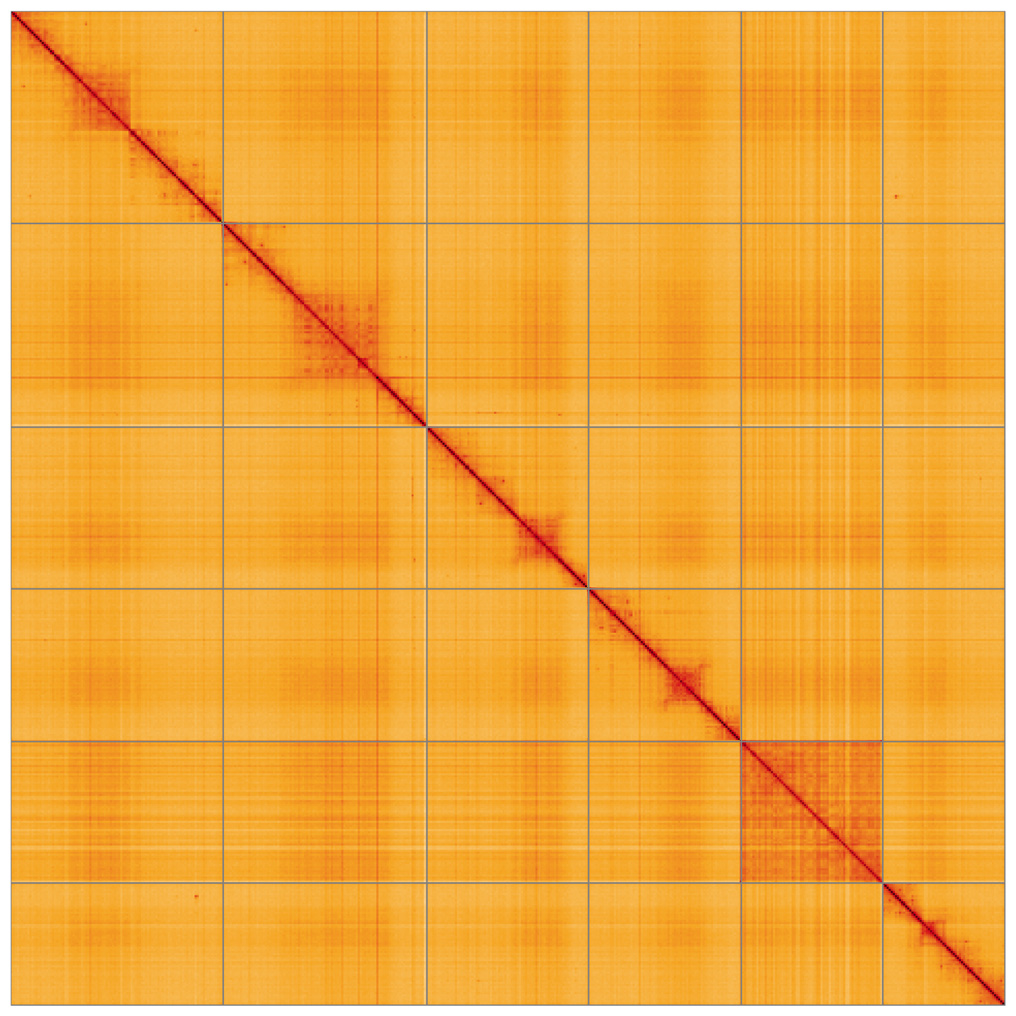
Genome assembly of *Merzomyia westermanni* idMerWest1.1: Hi-C contact map of the idMerWest1.1 assembly, visualised using HiGlass. Chromosomes are shown in order of size from left to right and top to bottom. An interactive version of this figure may be viewed at https://genome-note-higlass.tol.sanger.ac.uk/l/?d=FcOmBB_YR0GGS3J3zMpFLA.

Manual assembly curation corrected 131 missing joins or mis-joins and one haplotypic duplications, increasing the assembly length by 0.9%, and reducing the scaffold number by 79.78%. The final assembly has a total length of 986.20 Mb in 17 sequence scaffolds with a scaffold N50 of 160.3 Mb ( Table 2). The total count of gaps in the scaffolds is 241. The snail plot in Figure 2 provides a summary of the assembly statistics, while Figure 3 shows the distribution of base coverage against position for sequences in each chromosome of the assembly. The cumulative assembly plot in Figure 4 shows curves for subsets of scaffolds assigned to different phyla. Most (99.96%) of the assembly sequence was assigned to 6 chromosomal-level scaffolds. Chromosome-scale scaffolds confirmed by the Hi-C data are named in order of size ( Figure 5; Table 3). The sex chromosomes remain unidentified. While not fully phased, the assembly deposited is of one haplotype. Contigs corresponding to the second haplotype have also been deposited. The mitochondrial genome was also assembled and can be found as a contig within the multifasta file of the genome submission.

**Table 2.** Genome assembly data for *Merzomyia westermanni*, idMerWest1.1.

Genome assembly		
Assembly name	idMerWest1.1	
Assembly accession	GCA_949987695.1	
*Accession of alternate haplotype*	*GCA_950005075.1*	
Span (Mb)	986.20	
Number of contigs	259	
Contig N50 length (Mb)	9.3	
Number of scaffolds	17	
Scaffold N50 length (Mb)	160.3	
Longest scaffold (Mb)	210.65	
Assembly metrics *		*Benchmark*
Consensus quality (QV)	54.9	*≥ 50*
*k*-mer completeness	99.99%	*≥ 95%*
BUSCO **	C:97.5%[S:96.6%,D:0.9%], F:0.8%,M:1.6%,n:3,285	*C ≥ 95%*
Percentage of assembly mapped to chromosomes	99.96%	*≥ 95%*
Sex chromosomes	Not identified	*localised * *homologous pairs*
Organelles	Mitochondrial genome: 19.45 kb	*complete single * *alleles*
Genome annotation of assembly GCA_949987695.1 at Ensembl		
Number of protein-coding genes	25,765	
Number of gene transcripts	26,430	

* Assembly metric benchmarks are adapted from column VGP-2020 of “Table 1: Proposed standards and metrics for defining genome assembly quality” from Rhie *et al.* (2021). ** BUSCO scores based on the diptera_odb10 BUSCO set using version 5.3.2. C = complete [S = single copy, D = duplicated], F = fragmented, M = missing, n = number of orthologues in comparison. A full set of BUSCO scores is available at https://blobtoolkit.genomehubs.org/view/idMerWest1_1/dataset/idMerWest1_1/busco.

**Table 3.** Chromosomal pseudomolecules in the genome assembly of *Merzomyia westermanni*, idMerWest1.

INSDC accession	Name	Length (Mb)	GC%
OX465288.1	1	210.65	36.0
OX465289.1	2	201.96	37.0
OX465290.1	3	160.3	36.0
OX465291.1	4	151.49	36.5
OX465292.1	5	140.15	37.5
OX465293.1	6	121.24	36.0
OX465294.1	MT	0.02	20.0

The estimated Quality Value (QV) of the final assembly is 54.9 with *k*-mer completeness of 99.99%, and the assembly has a BUSCO v5.3.2 completeness of 97.5% (single = 96.6%, duplicated = 0.9%), using the diptera_odb10 reference set ( *n* = 3,285).

Metadata for specimens, BOLD barcode results, spectra estimates, sequencing runs, contaminants and pre-curation assembly statistics are given at https://links.tol.sanger.ac.uk/species/2795681.

## Genome annotation report

The *Merzomyia westermanni* genome assembly (GCA_949987695.1) was annotated at the European Bioinformatics Institute (EBI) on Ensembl Rapid Release. The resulting annotation includes 26,430 transcribed mRNAs from 25,765 protein-coding genes ( Table 2; https://rapid.ensembl.org/Merzomyia_westermanni_GCA_949987695.1/Info/Index). The average transcript length is 3,834.35. There are 1.03 coding transcripts per gene and 3.01 exons per transcript.

## Methods

### Sample acquisition

An adult *Merzomyia westermanni* (specimen ID Ox000750, ToLID idMerWest1) was collected from Wytham Woods, Oxfordshire (biological vice-county Berkshire), UK (latitude 51.77, longitude –1.33) on 2020-08-04 by netting. The specimen was collected and identified by Steven Falk (independent researcher) and preserved on dry ice. This specimen was used for PacBio HiFi and Illumina Hi-C sequencing. The specimen used for RNA sequencing (specimen ID Ox002808, ToLID idMerWest2) was an adult specimen netted in the same location on 2022-07-14. The specimen was collected by Steven Falk and Liam Crowley (University of Oxford), identified by Steven Falk, and then preserved on dry ice.

The initial identification was verified by an additional DNA barcoding process according to the framework developed by Twyford *et al*. (2024). A small sample was dissected from each specimen and stored in ethanol, while the remaining parts of the specimen were shipped on dry ice to the Wellcome Sanger Institute (WSI). The tissue was lysed, the COI marker region was amplified by PCR, and amplicons were sequenced and compared to the BOLD database, confirming the species identification ( Crowley *et al.*, 2023). Following whole genome sequence generation, the relevant DNA barcode region wass also used alongside the initial barcoding data for sample tracking at the WSI ( Twyford *et al.*, 2024). The standard operating procedures for Darwin Tree of Life barcoding have been deposited on protocols.io ( Beasley *et al.*, 2023).

### Nucleic acid extraction

The workflow for high molecular weight (HMW) DNA extraction at the WSI Tree of Life Core Laboratory includes a sequence of core procedures: sample preparation; sample homogenisation, DNA extraction, fragmentation, and clean-up. In sample preparation, the idMerWest1 sample was weighed and dissected on dry ice ( Jay *et al.*, 2023). Tissue from the thorax and abdomen was homogenised using a PowerMasher II tissue disruptor ( Denton *et al.*, 2023a).

HMW DNA was extracted using the Manual MagAttract v1 protocol ( Strickland *et al.*, 2023b). DNA was sheared into an average fragment size of 12–20 kb in a Megaruptor 3 system ( Todorovic *et al.*, 2023). Sheared DNA was purified by solid-phase reversible immobilisation ( Strickland *et al.*, 2023a): in brief, the method employs AMPure PB beads to eliminate shorter fragments and concentrate the DNA. The concentration of the sheared and purified DNA was assessed using a Nanodrop spectrophotometer and Qubit Fluorometer using the Qubit dsDNA High Sensitivity Assay kit. Fragment size distribution was evaluated by running the sample on the FemtoPulse system.

RNA was extracted from whole organism tissue of idMerWest2 in the Tree of Life Laboratory at the WSI using the RNA Extraction: Automated MagMax™ *mir*Vana protocol ( do Amaral *et al.*, 2023). The RNA concentration was assessed using a Nanodrop spectrophotometer and a Qubit Fluorometer using the Qubit RNA Broad-Range Assay kit. Analysis of the integrity of the RNA was done using the Agilent RNA 6000 Pico Kit and Eukaryotic Total RNA assay.

Protocols developed by the WSI Tree of Life laboratory are publicly available on protocols.io ( Denton *et al.*, 2023b).

### Sequencing

Pacific Biosciences HiFi circular consensus DNA sequencing libraries were constructed according to the manufacturers’ instructions. Poly(A) RNA-Seq libraries were constructed using the NEB Ultra II RNA Library Prep kit. DNA and RNA sequencing was performed by the Scientific Operations core at the WSI on Pacific Biosciences Sequel IIe (HiFi) and Illumina NovaSeq 6000 (RNA-Seq) instruments. Hi-C data were also generated from head tissue of idMerWest1 using the Arima-HiC v2 kit. The Hi-C sequencing was performed using paired-end sequencing with a read length of 150 bp on the Illumina NovaSeq 6000 instrument.

### Genome assembly, curation and evaluation

***Assembly***

The original assembly of HiFi reads was performed using Hifiasm ( Cheng *et al.*, 2021) with the --primary option. Haplotypic duplications were identified and removed with purge_dups ( Guan *et al.*, 2020). Hi-C reads were further mapped with bwa-mem2 ( Vasimuddin *et al.*, 2019) to the primary contigs, which were further scaffolded using the provided Hi-C data ( Rao *et al.*, 2014) in YaHS ( Zhou *et al.*, 2023) using the --break option. Scaffolded assemblies were evaluated using Gfastats ( Formenti *et al.*, 2022), BUSCO ( Manni *et al.*, 2021) and MERQURY.FK ( Rhie *et al.*, 2020).

The mitochondrial genome was assembled using MitoHiFi ( Uliano-Silva *et al.*, 2023), which runs MitoFinder ( Allio *et al.*, 2020) and uses these annotations to select the final mitochondrial contig and to ensure the general quality of the sequence.

***Assembly curation***

The assembly was decontaminated using the Assembly Screen for Cobionts and Contaminants (ASCC) pipeline (article in preparation). Manual curation was primarily conducted using PretextView ( Harry, 2022), with additional insights provided by JBrowse2 ( Diesh *et al.*, 2023) and HiGlass ( Kerpedjiev *et al.*, 2018). Scaffolds were visually inspected and corrected as described by Howe *et al*. (2021). Any identified contamination, missed joins, and mis-joins were corrected, and duplicate sequences were tagged and removed. The process is documented at https://gitlab.com/wtsi-grit/rapid-curation (article in preparation).

### Evaluation of the final assembly

A Hi-C map for the final assembly was produced using bwa-mem2 ( Vasimuddin *et al.*, 2019) in the Cooler file format ( Abdennur & Mirny, 2020). To assess the assembly metrics, the *k*-mer completeness and QV consensus quality values were calculated in Merqury ( Rhie *et al.*, 2020). This work was done using the “sanger-tol/readmapping” ( Surana *et al.*, 2023a) and “sanger-tol/genomenote” ( Surana *et al.*, 2023b) pipelines. The genome readmapping pipelines were developed using the nf-core tooling ( Ewels *et al.*, 2020), use MultiQC ( Ewels *et al.*, 2016), and make extensive use of the Conda package manager, the Bioconda initiative ( Grüning *et al.*, 2018), the Biocontainers infrastructure ( da Veiga Leprevost *et al.*, 2017), and the Docker ( Merkel, 2014) and Singularity ( Kurtzer *et al.*, 2017) containerisation solutions. The genome was also analysed within the BlobToolKit environment ( Challis *et al.*, 2020) and BUSCO scores ( Manni *et al.*, 2021; Simão *et al.*, 2015) were calculated.

Table 4 contains a list of relevant software tool versions and sources.

**Table 4.** Software tools: versions and sources.

Software tool	Version	Source
BlobToolKit	4.2.1	https://github.com/blobtoolkit/blobtoolkit
BUSCO	5.3.2	https://gitlab.com/ezlab/busco
bwa-mem2	2.2.1	https://github.com/bwa-mem2/bwa-mem2
Cooler	0.8.11	https://github.com/open2c/cooler
FreeBayes	1.3.1-17- gaa2ace8	https://github.com/freebayes/freebayes
Gfastats	1.3.6	https://github.com/vgl-hub/gfastats
Hifiasm	0.15.3	https://github.com/chhylp123/hifiasm
HiGlass	1.11.6	https://github.com/higlass/higlass
Long Ranger ALIGN	2.2.2	https://support.10xgenomics.com/genome-exome/ software/pipelines/latest/advanced/other-pipelines
Merqury	MerquryFK	https://github.com/thegenemyers/MERQURY.FK
MitoHiFi	2	https://github.com/marcelauliano/MitoHiFi
PretextView	0.2	https://github.com/wtsi-hpag/PretextView
purge_dups	1.2.3	https://github.com/dfguan/purge_dups
sanger-tol/ genomenote	v1.0	https://github.com/sanger-tol/genomenote
sanger-tol/ readmapping	1.1.0	https://github.com/sanger-tol/readmapping/tree/1.1.0
YaHS	1.0	https://github.com/c-zhou/yahs

### Genome annotation

The BRAKER2 pipeline ( Brůna *et al.*, 2021) was used in the default protein mode to generate annotation for the *Merzomyia westermanni* assembly (GCA_949987695.1) in Ensembl Rapid Release at the EBI.

### Wellcome Sanger Institute – Legal and Governance

The materials that have contributed to this genome note have been supplied by a Darwin Tree of Life Partner. The submission of materials by a Darwin Tree of Life Partner is subject to the **‘Darwin Tree of Life Project Sampling Code of Practice’**, which can be found in full on the Darwin Tree of Life website here. By agreeing with and signing up to the Sampling Code of Practice, the Darwin Tree of Life Partner agrees they will meet the legal and ethical requirements and standards set out within this document in respect of all samples acquired for, and supplied to, the Darwin Tree of Life Project.

Further, the Wellcome Sanger Institute employs a process whereby due diligence is carried out proportionate to the nature of the materials themselves, and the circumstances under which they have been/are to be collected and provided for use. The purpose of this is to address and mitigate any potential legal and/or ethical implications of receipt and use of the materials as part of the research project, and to ensure that in doing so we align with best practice wherever possible. The overarching areas of consideration are:

•     Ethical review of provenance and sourcing of the material

•     Legality of collection, transfer and use (national and international) 

Each transfer of samples is further undertaken according to a Research Collaboration Agreement or Material Transfer Agreement entered into by the Darwin Tree of Life Partner, Genome Research Limited (operating as the Wellcome Sanger Institute), and in some circumstances other Darwin Tree of Life collaborators.

## Data Availability

European Nucleotide Archive: Merzomyia westermanni. Accession number PRJEB48049; https://identifiers.org/ena.embl/PRJEB48049 ( Wellcome Sanger Institute, 2023). The genome sequence is released openly for reuse. The *Merzomyia westermanni* genome sequencing initiative is part of the Darwin Tree of Life (DToL) project. All raw sequence data and the assembly have been deposited in INSDC databases. Raw data and assembly accession identifiers are reported in Table 1 and Table 2.

## Author information

Members of the University of Oxford and Wytham Woods Genome Acquisition Lab are listed here: https://doi.org/10.5281/zenodo.12157525.

Members of the Darwin Tree of Life Barcoding collective are listed here: https://doi.org/10.5281/zenodo.12158331

Members of the Wellcome Sanger Institute Tree of Life Management, Samples and Laboratory team are listed here: https://doi.org/10.5281/zenodo.12162482.

Members of Wellcome Sanger Institute Scientific Operations: Sequencing Operations are listed here: https://doi.org/10.5281/zenodo.12165051.

Members of the Wellcome Sanger Institute Tree of Life Core Informatics team are listed here: https://doi.org/10.5281/zenodo.12160324.

Members of the Tree of Life Core Informatics collective are listed here: https://doi.org/10.5281/zenodo.12205391.

Members of the Darwin Tree of Life Consortium are listed here: https://doi.org/10.5281/zenodo.4783558.

## References

[ref-1] Abdennur N Mirny LA : Cooler: scalable storage for Hi-C data and other genomically labeled arrays. *Bioinformatics.* 2020;36(1):311–316. 10.1093/bioinformatics/btz540 31290943 PMC8205516

[ref-2] Allio R Schomaker-Bastos A Romiguier J : MitoFinder: efficient automated large-scale extraction of mitogenomic data in target enrichment phylogenomics. *Mol Ecol Resour.* 2020;20(4):892–905. 10.1111/1755-0998.13160 32243090 PMC7497042

[ref-3] Beasley J Uhl R Forrest LL : DNA barcoding SOPs for the Darwin Tree of Life project. *protocols.io.* 2023; [Accessed 25 June 2024]. 10.17504/protocols.io.261ged91jv47/v1

[ref-4] Brůna T Hoff KJ Lomsadze A : BRAKER2: automatic eukaryotic genome annotation with GeneMark-EP+ and AUGUSTUS supported by a protein database. *NAR Genom Bioinform.* 2021;3(1): lqaa108.1–11. 10.1093/nargab/lqaa108 33575650 PMC7787252

[ref-45] Challis R Richards E Rajan J : BlobToolKit – interactive quality assessment of genome assemblies. *G3 (Bethesda).* 2020;10(4):1361–1374. 10.1534/g3.119.400908 32071071 PMC7144090

[ref-5] Cheng H Concepcion GT Feng X : Haplotype-resolved *de novo* assembly using phased assembly graphs with hifiasm. *Nat Methods.* 2021;18(2):170–175. 10.1038/s41592-020-01056-5 33526886 PMC7961889

[ref-6] Coldwell JD : *Merzomyia westermanni*(Meigen) (Diptera, Tephritidae) new to Yorkshire. *Dipterists Digest.* 2000;7(2): 102.

[ref-7] Crowley L Allen H Barnes I : A sampling strategy for genome sequencing the British terrestrial arthropod fauna [version 1; peer review: 2 approved]. *Wellcome Open Res.* 2023;8:123. 10.12688/wellcomeopenres.18925.1 37408610 PMC10318377

[ref-8] da Veiga Leprevost F Grüning BA Alves Aflitos S : BioContainers: an open-source and community-driven framework for software standardization. *Bioinformatics.* 2017;33(16):2580–2582. 10.1093/bioinformatics/btx192 28379341 PMC5870671

[ref-9] Denton A Oatley G Cornwell C : Sanger Tree of Life sample homogenisation: PowerMash. *protocols.io.* 2023a. 10.17504/protocols.io.5qpvo3r19v4o/v1

[ref-10] Denton A Yatsenko H Jay J : Sanger Tree of Life wet laboratory protocol collection V.1. *protocols.io.* 2023b. 10.17504/protocols.io.8epv5xxy6g1b/v1

[ref-11] Diesh C Stevens GJ Xie P : JBrowse 2: a modular genome browser with views of synteny and structural variation. *Genome Biol.* 2023;24(1): 74. 10.1186/s13059-023-02914-z 37069644 PMC10108523

[ref-12] do Amaral RJV Bates A Denton A : Sanger Tree of Life RNA extraction: automated MagMax ^TM^ mirVana. *protocols.io.* 2023. 10.17504/protocols.io.6qpvr36n3vmk/v1

[ref-14] Ewels P Magnusson M Lundin S : MultiQC: summarize analysis results for multiple tools and samples in a single report. *Bioinformatics.* 2016;32(19):3047–3048. 10.1093/bioinformatics/btw354 27312411 PMC5039924

[ref-13] Ewels PA Peltzer A Fillinger S : The nf-core framework for community-curated bioinformatics pipelines. *Nat Biotechnol.* 2020;38(3):276–278. 10.1038/s41587-020-0439-x 32055031

[ref-15] Falk SJ : Some noteworthy records and observations of tephritid flies. *Dipterists Digest.* 1991;8:32–35.

[ref-16] Falk SJ Ismay JW Chandler PJ : A provisional assessment of the status of Acalyptratae flies in the UK. *Natural England Commissioned Reports 217*.2016. Reference Source

[ref-17] Formenti G Abueg L Brajuka A : Gfastats: conversion, evaluation and manipulation of genome sequences using assembly graphs. *Bioinformatics.* 2022;38(17):4214–4216. 10.1093/bioinformatics/btac460 35799367 PMC9438950

[ref-18] GBIF.org: GBIF Occurrence Download *Merzomyia westermanni* (Meigen, 1826).2024; [Accessed 3 July 2024]. Reference Source

[ref-19] Grüning B Dale R Sjödin A : Bioconda: sustainable and comprehensive software distribution for the life sciences. *Nat Methods.* 2018;15(7):475–476. 10.1038/s41592-018-0046-7 29967506 PMC11070151

[ref-20] Guan D McCarthy SA Wood J : Identifying and removing haplotypic duplication in primary genome assemblies. *Bioinformatics.* 2020;36(9):2896–2898. 10.1093/bioinformatics/btaa025 31971576 PMC7203741

[ref-21] Harry E : PretextView (Paired REad TEXTure Viewer): a desktop application for viewing pretext contact maps.2022; [Accessed 19 October 2022]. Reference Source

[ref-22] Howe K Chow W Collins J : Significantly improving the quality of genome assemblies through curation. *GigaScience.* 2021;10(1): giaa153. 10.1093/gigascience/giaa153 33420778 PMC7794651

[ref-23] Jay J Yatsenko H Narváez-Gómez JP : Sanger Tree of Life sample preparation: triage and dissection. *protocols.io.* 2023. 10.17504/protocols.io.x54v9prmqg3e/v1

[ref-24] Kerpedjiev P Abdennur N Lekschas F : HiGlass: web-based visual exploration and analysis of genome interaction maps. *Genome Biol.* 2018;19(1): 125. 10.1186/s13059-018-1486-1 30143029 PMC6109259

[ref-25] Kurtzer GM Sochat V Bauer MW : Singularity: scientific containers for mobility of compute. *PLoS One.* 2017;12(5): e0177459. 10.1371/journal.pone.0177459 28494014 PMC5426675

[ref-26] Manni M Berkeley MR Seppey M : BUSCO update: novel and streamlined workflows along with broader and deeper phylogenetic coverage for scoring of eukaryotic, prokaryotic, and viral genomes. *Mol Biol Evol.* 2021;38(10):4647–4654. 10.1093/molbev/msab199 34320186 PMC8476166

[ref-27] Merkel D : Docker: lightweight Linux containers for consistent development and deployment. *Linux J.* 2014;2014(239): 2. Reference Source

[ref-28] Miranda C Cheeke P Schmitz J : Toxicity of *Senecio jacobaea* (Tansy Ragwort) in rats. *Toxicol Appl Pharmacol.* 1980;56(3):431–442. 10.1016/0041-008x(80)90077-0 7222027

[ref-29] Rao SSP Huntley MH Durand NC : A 3D map of the human genome at kilobase resolution reveals principles of chromatin looping. *Cell.* 2014;159(7):1665–1680. 10.1016/j.cell.2014.11.021 25497547 PMC5635824

[ref-30] Rhie A McCarthy SA Fedrigo O : Towards complete and error-free genome assemblies of all vertebrate species. *Nature.* 2021;592(7856):737–746. 10.1038/s41586-021-03451-0 33911273 PMC8081667

[ref-31] Rhie A Walenz BP Koren S : Merqury: reference-free quality, completeness, and phasing assessment for genome assemblies. *Genome Biol.* 2020;21(1): 245. 10.1186/s13059-020-02134-9 32928274 PMC7488777

[ref-32] Seguy E : Dipteres (Brachyceres): Muscidae Acalyptera et Scatophagidae Faune de France 28.Paris: Paul Lechevalier.1934. Reference Source

[ref-46] Simão FA Waterhouse RM Ioannidis P : BUSCO: assessing genome assembly and annotation completeness with single-copy orthologs. *Bioinformatics.* 2015;31(19):3210–3212. 10.1093/bioinformatics/btv351 26059717

[ref-33] Strickland M Cornwell C Howard C : Sanger Tree of Life fragmented DNA clean up: manual SPRI. *protocols.io.* 2023a. 10.17504/protocols.io.kxygx3y1dg8j/v1

[ref-34] Strickland M Moll R Cornwell C : Sanger Tree of Life HMW DNA extraction: manual MagAttract. *protocols.io.* 2023b. 10.17504/protocols.io.6qpvr33novmk/v1

[ref-35] Surana P Muffato M Qi G : sanger-tol/readmapping: sanger-tol/readmapping v1.1.0 - Hebridean Black (1.1.0). *Zenodo.* 2023a. 10.5281/zenodo.7755669

[ref-36] Surana P Muffato M Sadasivan Baby C : sanger-tol/genomenote (v1.0.dev). *Zenodo.* 2023b. 10.5281/zenodo.6785935

[ref-37] Todorovic M Sampaio F Howard C : Sanger Tree of Life HMW DNA fragmentation: diagenode Megaruptor ^®^3 for PacBio HiFi. *protocols.io.* 2023. 10.17504/protocols.io.8epv5x2zjg1b/v1

[ref-38] Twyford AD Beasley J Barnes I : A DNA barcoding framework for taxonomic verification in the Darwin Tree of Life project [version 1; peer review: awaiting peer review]. *Wellcome Open Res.* 2024;9:339. 10.12688/wellcomeopenres.21143.1 PMC11462125 39386966

[ref-39] Uliano-Silva M Ferreira JGRN, Krasheninnikova K : MitoHiFi: a python pipeline for mitochondrial genome assembly from PacBio high fidelity reads. *BMC Bioinformatics.* 2023;24(1): 288. 10.1186/s12859-023-05385-y 37464285 PMC10354987

[ref-40] Vasimuddin M Misra S Li H : Efficient architecture-aware acceleration of BWA-MEM for multicore systems.In: *2019 IEEE International Parallel and Distributed Processing Symposium (IPDPS).*IEEE,2019;314–324. Reference Source

[ref-41] Watt TA : The biology and toxicity of ragwort ( *Senecio jacobaea* L.) and its herbicidal and biological control. *Herb Abstr.* 1987;57(1):1–16. Reference Source

[ref-44] Wellcome Sanger Institute: The genome sequence of a tephritid fruit fly, *Merzomyia westermanni* Meigen 1826. European Nucleotide Archive.[dataset], accession number PRJEB48049,2023.

[ref-42] White IM : Tephritid flies: Diptera: Tephritidae, 1.In: *Handbooks for the Identification of British Insects.*London: Royal Entomological Society of London,1988. Reference Source

[ref-43] Zhou C McCarthy SA Durbin R : YaHS: yet another Hi-C scaffolding tool. *Bioinformatics.* 2023;39(1): btac808. 10.1093/bioinformatics/btac808 36525368 PMC9848053

