# Supplementary material for: The genome sequence of a tephritid fruit fly, Merzomyia westermanni Meigen 1826
Source: Wellcome Open Res. 2024 Aug 14;9:480. [Version 1] doi: 10.12688/wellcomeopenres.22873.1 (PMC11462123; doi:10.12688/wellcomeopenres.22873.1)
Supplement: Peer review history

# Reviewer report: Reviewer response for version 1

**DOI:** 10.21956/wellcomeopenres.25188.r100314

**Published:** 2024-10-08

**Authors:** Jesus Lozano-Fernandez

**Affiliations:** 1 Universitat de Barcelona, Barcelona, Spain

This manuscript presents a chromosome-scale genome assembly based on a single individual of *Merzomyia westermanni*, a tephritid fruit fly. The methods employed to infer the assembly are up-to-date and rigorous, and the final assembly presented is based on a sound analyses of the data. I do not have any major remarks to this article, just a very minor comments.

 In the Background section, where addressing the interaction of this insect and a plant, there is likely a typo for the genus " *Senecio*". Regarding sequencing depth and different technologies employed (PacBio long reads, Hi-C Illumina, Illumina short-reads and Illumina RNA-seq), they seem enough to retrieve a complete genome (always >20x). The inferred 6 chromosome-levels scaffolds contains most of the sequences and are mostly assigned to Arthropoda and BUSCO completeness is high, which suggests that the genome assembly is highly-complete and free of contamination. The authors provide the assembly for one of the haplotypes of this diploid organism, and a snail plot figure depicting most assembly of metrics.  There is a typo in Methods sections "wass".

Are sufficient details of methods and materials provided to allow replication by others?

Yes

Is the rationale for creating the dataset(s) clearly described?

Yes

Are the datasets clearly presented in a useable and accessible format?

Yes

Are the protocols appropriate and is the work technically sound?

Yes

Reviewer Expertise:

Arthropods, phylogenomics, comparative genomics, terrestrialization, biospeleology

I confirm that I have read this submission and believe that I have an appropriate level of expertise to confirm that it is of an acceptable scientific standard.

# Reviewer report: Reviewer response for version 1

**DOI:** 10.21956/wellcomeopenres.25188.r99359

**Published:** 2024-09-27

**Authors:** Ke-Long Jiao

**Affiliations:** 1 Tianjin Agricultural University, Tianjin, Tianjin, China

The introduction of the article briefly outlines the fundamental taxonomic status, ecological characteristics, geographical distribution, and current research status of *Merzomyia westermanni*, laying a solid foundation for subsequent genomic sequencing and analysis. It is recommended to incorporate more discussions on the role of this species in the ecosystem, its evolutionary relationships with other species, and so forth, to enrich the depth and breadth of the article.

 The Methods section meticulously details the entire process of sample collection, genomic sequencing, assembly, and annotation, showcasing the rigor and systematic approach of the scientific research. To enhance the clarity and reproducibility of the experiments, the authors could further elaborate on specific operational details of certain steps, such as the exact parameters used during the assembly process and the advancements or uniqueness of the methods employed. This would not only reflect the authors' insights gained during the experimental process but also allow readers to gain a more comprehensive understanding of the experimental procedures.

 The Results section presents comprehensive data on the genome of *Merzomyia westermanni*, encompassing crucial information like genome size, chromosome structure, and gene count. In the Results Analysis, the authors could delve deeper into the biological implications of the genome by comparing gene differences with closely related species, exploring the presence of specific gene families, and identifying potential host-interaction genes. Such endeavors would enrich the content and depth of the article.

 The Discussion section succinctly summarizes the key findings of the study and highlights the potential applications of the genomic data in species conservation, pest control, and other areas. This section could be expanded to delve into how the genomic data can contribute to solving economic challenges in animal husbandry, as well as addressing pressing issues in ecology and genetics. Additionally, sharing insights into future research directions and achievable goals based on the genome would provide a forward-looking perspective.

Are sufficient details of methods and materials provided to allow replication by others?

Yes

Is the rationale for creating the dataset(s) clearly described?

Yes

Are the datasets clearly presented in a useable and accessible format?

Yes

Are the protocols appropriate and is the work technically sound?

Yes

Reviewer Expertise:

Insect taxonomists, insect genomicists, and experts in insect phylogenetic studies.

I confirm that I have read this submission and believe that I have an appropriate level of expertise to confirm that it is of an acceptable scientific standard.
